# Clinically important left superior polar artery giving rise to a left inferior suprarenal artery and an aberrant left inferior polar artery: a case report

**DOI:** 10.1590/1677-5449.202300122

**Published:** 2023-06-30

**Authors:** Naveen Kumar, Ashwini Aithal Padur, Mohandas Rao Kappettu Gadahad, Swamy Ravindra Shanthakumar

**Affiliations:** 1 RAK Medical & Health Sciences University – RAKMHSU, RAK College of Medical Sciences, Ras-Al-Khaimah, United Arab Emirates.; 2 Manipal Academy of Higher Education, Manipal, Karnataka, India.

**Keywords:** renal artery, polar artery, inferior suprarenal artery, ureter, inferior mesenteric vein, artéria renal, artéria polar, artéria suprarrenal inferior, ureter, veia mesentérica inferior

## Abstract

The renal arteries arise from the lateral side of the abdominal aorta at the L2 vertebral level, just below the origin of the superior mesenteric artery. Multiple aberrant renal arteries can pose difficulties in renal transplantation, interventional radiological and urological procedures, renal artery embolization, angioplasty, or vascular reconstruction for congenital and acquired lesions. We present a case of a left kidney supplied by the left renal artery along with superior and inferior polar arteries, arising from the aorta and inferior mesenteric artery respectively. The inferior mesenteric artery was crossed by the left ureter and inferior mesenteric vein. The superior polar artery gave rise to an inferior suprarenal artery making the variation important for clinicians and surgeons.

## Resumo

As artérias renais originam-se do lado lateral da aorta abdominal, no nível da vértebra L2, logo abaixo da origem da artéria mesentérica superior. A presença de múltiplas artérias renais aberrantes pode representar dificuldades para transplante renal, procedimentos radiológicos e urológicos intervencionistas, embolização da artéria renal, angioplastia e reconstrução vascular para lesões congênitas e adquiridas. Apresentamos um caso de rim esquerdo vascularizado pela artéria renal esquerda e pelas artérias polares superior e inferior, as quais se originavam da aorta e da artéria mesentérica inferior, respectivamente. A artéria mesentérica inferior era cruzada pelo ureter esquerdo e pela veia mesentérica inferior. A artéria polar superior dava origem à artéria suprarrenal inferior, o que torna essa variação importante para clínicos e cirurgiões.

## INTRODUCTION

Knowledge of aberrant renal arterial systems is important for surgeons and clinicians to avoid complications during renal transplants, interventional radiological procedures, and renal vascular operations. Even though numerous variations of renal vasculature have been observed and reported, new variations can pose difficulties to clinicians during surgical procedures if not discovered before commencing the procedure. Aberrant renal arteries can be present as polar arteries or as accessory or hilar arteries. Polar arteries supply the superior or inferior poles of the kidneys without entering the renal hilum, while hilar arteries enter the renal hilum to supply the kidney. Polar arteries may originate from the aorta or common iliac arteries and may rarely originate from the suprarenal artery, the celiac trunk, superior mesenteric, inferior mesenteric, median sacral, or phrenic arteries.^1^ These polar arteries represent persistent fetal mesonephric branches of the dorsal aorta.^2^ The renal arteries arise from the lateral side of the abdominal aorta at the L2 vertebral level, just below the origin of the superior mesenteric artery. The renal artery traverses towards the medial border of its corresponding kidney and lies between the renal vein anteriorly and the renal pelvis posteriorly at the renal hilum. At the hilum, the renal artery divides into anterior and posterior branches that further divide into segmental branches.^3^

## CASE REPORT

Aberrant left renal arteries were observed during dissection of the abdomen for undergraduate medical students in an elderly male cadaver. No medical history was available on this cadaver. This study is in compliance with the Helsinki Declaration and with local ethical guidelines.

**Figure 1:**
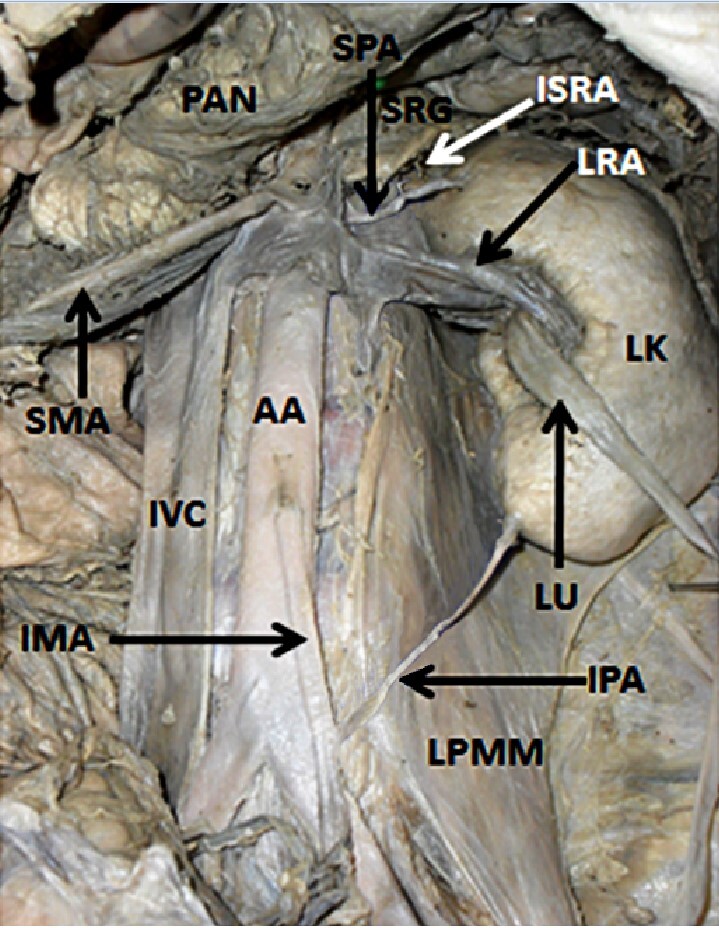
Dissection of abdomen showing retracted left ureter (LU) and the left kidney (LK) supplied by the left renal artery (LRA), a superior polar artery (SPA), and an inferior polar artery (IPA), arising from the abdominal aorta (AA) and inferior mesenteric artery (IMA) respectively. The inferior suprarenal artery (ISRA) can be seen emerging from the superior polar artery. LPMM – left psoas major muscle, IVC – inferior vena cava, SMA – superior mesenteric artery, SRG – suprarenal gland, PAN – pancreas.

The left kidney was supplied by superior and inferior polar arteries in addition to the left renal artery. The superior polar artery was a direct lateral branch of the abdominal aorta, taking its origin at the level of the origin of the superior mesenteric artery and supplying the upper pole of the left kidney. It measured about 4.6 cm in length. While passing towards the left kidney, the superior artery gave rise to an inferior suprarenal artery. This inferior suprarenal artery ascended cranially and supplied the left suprarenal gland. The inferior polar artery took its origin as a branch from the inferior mesenteric artery (Figure 1), ascended cranially, passing behind the left ureter and inferior mesenteric vein, and entered the left kidney near its lower pole. It was about 13.8 cm in length. In addition to this, it was observed that the left renal vein received a left lumbar vein as a tributary. The multiple variations reported here were observed unilaterally on the left side.

## DISCUSSION

Polar arteries have been observed with variable incidences in studies regarding aberrant renal vessels. Sampaio and Passos^4^ dissected 266 kidneys in a study concerning renal arteries and mentioned that a single renal artery was observed in 53.3% of cases. One hilar and one superior polar artery were present in 14.3% cases, a superior polar artery was seen in 6.8%, and an inferior polar artery was found in 5.3% of cases.^4^

A study by Budhiraja et al.^5^ found a superior polar renal artery present in 22.6% of cases of cadaveric kidneys observed. This polar artery branched from the abdominal aorta in 10.7% of cases, was a direct branch from the main renal artery in 5.4% of cases, a branch from the superior hilar renal artery in 3.6% of cases, and a segmental branch from the renal artery in 3.0% of cases.^5^ As described by Palmieri et al.,^1^ there were multiple arteries in 61.5% of renal pedicles. An aortic origin of the multiple arteries was more frequent on the right side. In another study, one superior polar artery was observed on the right side (7.14%) or the left side (11.6%) with presence of one inferior polar on the right side (3.57%) or left side (2.9%).^1^ In the present case, the aberrant superior polar artery and the inferior polar artery were on the left side. According to Chauhan et al.,^6^ anomalies of renal arteries were found in 20 (50%) out of 40 formalin fixed cadavers. Out of 80 kidney specimens, a superior polar artery was observed in 10 (12.5%) kidneys and an inferior polar artery was observed in 10 (12.5%) kidneys. It was also noted that all superior polar arteries arose from the abdominal aorta at the level of the superior mesenteric artery and all inferior polar arteries arose from the abdominal aorta, except in one cadaver in which it arose from the renal artery.^6^ The percentages of superior polar arteries and inferior polar arteries were 6.67% and 10% of aberrant renal arteries respectively, as revealed by a study by Ankolekar and Sengupta.^7^ The percentages of origin from the aorta of superior polar arteries and inferior polar arteries were 3.33% and 8.33% respectively and the proportion of origin from main renal arteries was 2.33%.^7^ Anatomical findings from 267 Thai cadavers revealed one hilar artery with an upper polar artery in 7%; one hilar artery combined with one lower polar artery in 3%; two hilar arteries with one upper polar artery in 0.4%; and two hilar arteries with one lower polar artery in 0.6%.^8^

Shakeri et al.^9^ reported a case of a left accessory renal artery originating from the aorta and supplying both upper and lower renal poles through polar arteries.^9^ In the present case, the inferior polar artery originated from the inferior mesenteric artery and the superior polar artery was a branch from the aorta. A case report by Patil and Mishra^10^ describes an inferior polar renal artery arising from the anterolateral aspect of the aorta, passing superficial to the ureter and testicular vein on the right side, and involving possible obstruction of the ureter or testicular vein concerned, which should be diagnosed prior to surgical treatment of hydronephrosis or varicocele.^10^ Inferior polar arteries are found in 9% of the population. Some cases of inferior polar arteries can be responsible for vascular obstruction in the pelviureteric syndrome, which makes imaging essential.^11^ A case of ureteropelvic junction obstruction causing mild left hydronephrosis was detected in a 13-year-old boy and was found to be due to crossing of accessory renal artery or inferior polar artery anterior to the ureter,^12^ whereas in the present case the inferior polar artery passed posterior to the ureter.

Knowledge of the arterial and venous vascularization of the suprarenal gland is important in angiographic studies to identify benign or malignant and functioning or non-functioning lesions of the adrenal gland. The possibility of a variant origin of the inferior suprarenal artery should be kept in mind before an angiographic study or adrenalectomy in case of carcinomas, neuroblastomas, and pheochromocytomas since it could be a source of bleeding during surgery and in the postoperative period.^13^

In the present case, the left inferior polar artery may itself have been compressed by the ureter, leading to decreased blood supply to the inferior pole of the left kidney. The present case is unique as the left kidney was supplied by three separate arteries, i.e., the left renal artery, a left superior polar artery from the aorta, and a left inferior polar artery from the inferior mesenteric artery. Moreover, the superior polar artery gave rise to an inferior suprarenal artery, which makes this case unique and such a combination of aberrant arteries has not been reported earlier. During renal transplant, an aberrant renal vasculature of the donor kidney could pose difficulties. Thus, for renal transplantation and interventional radiological and urological procedures, renal artery embolization, angioplasty, and vascular reconstruction for congenital and acquired lesions,^6^ renal variations as in the present case are important for radiologists, clinicians, and surgeons.
